# Gene expression changes in human mesenchymal stem cells from patients with osteoporosis

**DOI:** 10.3892/mmr.2015.3514

**Published:** 2015-03-19

**Authors:** LIANYONG LIU, QINGYUN ZHU, JINGNAN WANG, QIAN XI, HONGLING ZHU, MINGJUN GU

**Affiliations:** 1 Department of Endocrinology, Shanghai Pudong Gongli Hospital, Shanghai 200135, P.R. China; 2 Department of Gastroenterology, Shanghai Pudong Gongli Hospital, Shanghai 200135, P.R. China

**Keywords:** osteoporosis, pathway analysis, transcriptional regulatory network

## Abstract

The aim of the present study was to investigate the underlying molecular mechanisms of osteoporosis and to identify novel candidate genes involved in this disease. The gene expression profile of GSE35958 was downloaded from Gene Expression Omnibus, including five samples of human mesenchymal stem cells from patients with osteoporosis and four control samples. Differentially expressed genes (DEGs) were initially identified following an analysis using Student’s t-test. Subsequently, a protein-protein interaction (PPI) network of the significant pathways was constructed, based on the Human Protein Reference Database. In the significant pathways, DEGs were screened using cut-off criteria of FDR<0.1 and |log_2_FC|>1.5. A co-change network for pathways was also constructed using the method of cumulative hypergeometric probability distribution. Finally, the transcriptional regulatory network for DEGs was constructed based on the TRANSFAC database. In total, 1,127 DEGs, including 554 upregulated and 573 downregulated DEGs, were screened. The constructed PPI network for the DEGs involved in the two significant pathways, including focal adhesion and lysosome, demonstrated that the five DEGs with a high degree (>60) were β-catenin, SHC-transforming protein 1, RAC-α serine/threonine-protein kinase, caveolin 1 and filamin A, with degrees of 135, 117, 117, 73 and 63, respectively. The pathway with the degree of 22 in the constructed co-change network was neuroactive ligand receptor interaction. The nine genes with a high (≥9) degree in the constructed transcriptional regulatory network were REL-associated protein, upstream stimulatory factor 1, specificity protein 1, Fos-related antigen 1, cyclin-dependent kinase inhibitor 1A, upstream stimulatory factor 2, ETS domain-containing protein Elk-1, JUND and retinoic acid receptor α, with degrees of 29, 27, 19, 18, 17, 13, 11, 11 and 9, respectively. The DEGs with high degree in the PPI and transcriptional regulatory networks may be candidate target molecules, which may be used to monitor, diagnose and treat osteoporosis.

## Introduction

Osteoporosis, characterized by the loss of bone mass and strength, and the development of microarchitecture impairment leading to fragility fractures, has become a significant clinical problem in health care services dealing with aging populations (1,2). The susceptibility to osteoporosis is regulated by a variety of factors, such as genetic variants, age, sex steroid production, lifestyle and environment (3–5).

A number of studies have investigated the pathogenesis of osteoporosis at the molecular levels. Two cytokines, including osteoprotegerin and receptor activator of nuclear factor κB ligand, have been identified as important regulators in the development of osteoporosis (2,6). Members of the Wnt signaling pathway, such as low-density lipoprotein receptor-related protein 5 (LRP5), Wnt3a, secreted Frizzled-related protein 1 and sclerostin (SOST), have been reported to be associated with variation in bone mineral density (7). Additionally, Wnt signaling may enhance osteoblast survival, and interact with parathyroid hormone signaling and bone morphogenetic protein 2, leading to an elevation in osteoblastogenesis (8–10). The transcription factor, specificity protein 1 (Sp1), is associated with a reduction in bone quality and the biomechanical properties of bone (11). However, the underlying etiology of osteoporosis is not yet comprehensively understood and the identification of novel therapeutic targets for osteoporosis is required.

Mesenchymal stem cells (MSCs) from bone marrow are multipotent cells that are able to differentiate into multiple cell lineages, including osteoblasts, adipocytes, fibroblasts and chondrocytes (12,13). The implantation of MSCs has been shown to be an effective and safe method by which to enhance bone regeneration and repair in animal models for bone regeneration as well as in clinical practice (14,15). Gene-expression microarrays are a powerful tool with high-throughput technology, which may be used to assess the expression patterns of multiple genes simultaneously. Therefore, gene expression microarray analysis of MSCs from patients with osteoporosis, may provide novel insights into the mechanisms underlying the pathogenesis of osteoporosis.

In the present study, gene expression profiles of MSCs from patients with osteoporosis and controls were downloaded, in order to identify differentially expressed genes (DEGs). The screened DEGs were further analyzed using bioinformatics methods to reveal osteoporosis-specific gene expression patterns. The aim was to provide novel targets for the diagnosis and treatment of osteoporosis.

## Materials and methods

### Samples and data preprocessing

The gene expression profile of GSE35958 (16) was downloaded from the National Center of Biotechnology Information Gene Expression Omnibus (GEO, http://www.ncbi.nlm.nih.gov/geo/), including five samples of human MSCs from the femoral heads of elderly patients with osteoporosis and four control bone marrow samples from age-matched non-osteoporotic donors. The platform used was GPL570 [HG-U133_Plus_2] Affymetrix Human Genome U133 Plus 2.0 Array (Affymetrix UK Ltd, High Wycombe, United Kingdom).

The downloaded data in CEL files was preprocessed using the Affy package. Background correction and quartile data normalization were performed using the robust multiarray average algorithm (17). Probes without a corresponding gene symbol were then filtered and the average value of gene symbols with multiple probes was calculated. Finally, the expression profile dataset, including 20,539 genes for the nine samples, was obtained.

### Screening DEGs

Student’s t-test was used to identify DEGs between the osteoporosis and control samples. The Benjamini-Hochberg (BH) procedure (18) was used to adjust the raw P-values into false discovery rates (FDRs). The DEGs were screened using cut-off criteria of FDR<0.1 and |log_2_FC|>1.5.

### Functional and pathway enrichment analysis for DEGs

In order to identify biological functions associated with the pathogenesis of osteoporosis, Gene Ontology (GO) (19) functional and Kyoto Encyclopedia of Genes and Genomes (KEGG) (20) pathway enrichment analyses were performed for the identified DEGs, using the online tool of Database for Annotation, Visualization and Integrated Discovery (DAVID) (21) based on the method of Expression Analysis Systemic Explorer (EASE) test (22). The enrichment threshold was an EASE score of 0.1.

### Construction of the protein-protein interaction network

Following the acquisition of pathways in which the DEGs with FDR<0.1 and |log_2_FC|>1.5 were markedly enriched, a protein-protein interaction (PPI) network of the significant pathways was constructed, based on the Human Protein Reference Database (HPRD) (23). DEGs, which may convey effective information regarding the pathogenesis of osteoporosis in the constructed PPI network were identified.

### Construction of co-change network for pathways

The co-change network for pathways was established based on the method of cumulative hypergeometric probability distribution (24). The pathway-pathway interactions with P<0.01 were identified to construct the co-change network for pathways. P-values were calculated using the following formula:

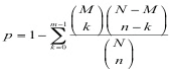
Where N is the total number of protein-protein interactions involved with DEGs, M is the number of protein-protein interactions associated with DEGs in a pathway, n is the number of protein-protein interactions involved with DEGs in other pathways and k is the number of protein-protein interactions involved with DEGs between the two pathways.

### Establishment of transcriptional regulatory network for DEGs

TRANSFAC (25) is a database containing information on eukaryotic transcription regulating DNA sequence elements, their genomic binding sites and their DNA-binding profiles. The transcriptional regulatory network for DEGs was constructed based on the TRANSFAC database.

## Results

### Identification of differentially expressed genes

Student’s t-test and the BH procedure were used to identify DEGs between the osteoporosis and control samples. A total of 1,127 DEGs were identified, with the cut-off criteria of FDR<0.1 and |log_2_FC|>1.5, including 554 upregulated and 573 downregulated DEGs.

### Functional and pathway enrichment analysis for DEGs

The screened DEGs were used for functional and pathway enrichment analysis by DAVID. A total of 27 DEGs had significant involvement in the hsa04510 pathway (focal adhesion; FDR=0.0205) and 17 DEGs were significantly enriched in the hsa04142 pathway (lysosome; FDR= 0.0477).

### Protein-protein interaction network construction

**Figure 1:**
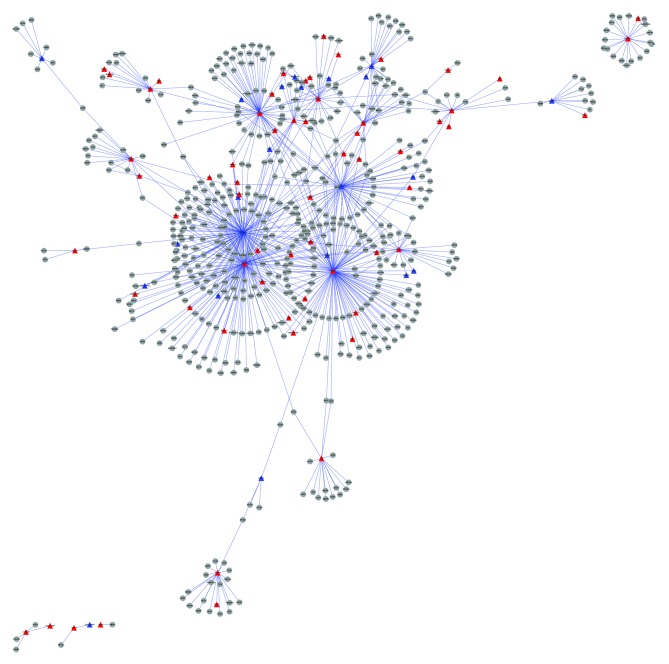
Protein-protein interaction network for DEGs involved in significantly enriched pathways. The red, blue and grey nodes, and grey edges represent the upregulated DEGs, downregulated DEGs, other genes and protein-protein interactions, respectively. DEGs, differentially expressed genes.

The protein-protein interactions for DEGs involved in the two significant pathways, including focal adhesion and lysosome, were identified, and were used to construct a PPI network (Fig. 1). Table I shows DEGs with a degree >60 in the constructed PPI network, including β-catenin (CTNNB1, 135), SHC-transforming protein 1 (SHC1, 117), RAC-α serine/thre-onine-protein kinase (AKT1, 117), caveolin 1 (CAV1, 73) and filamin A (FLNA, 63). CTNNB1 and CAV1 were significantly downregulated in the samples from the patients with osteoporosis, while the other three genes were upregulated (Table I).

### Establishment of a co-change network for pathways

**Figure 2:**
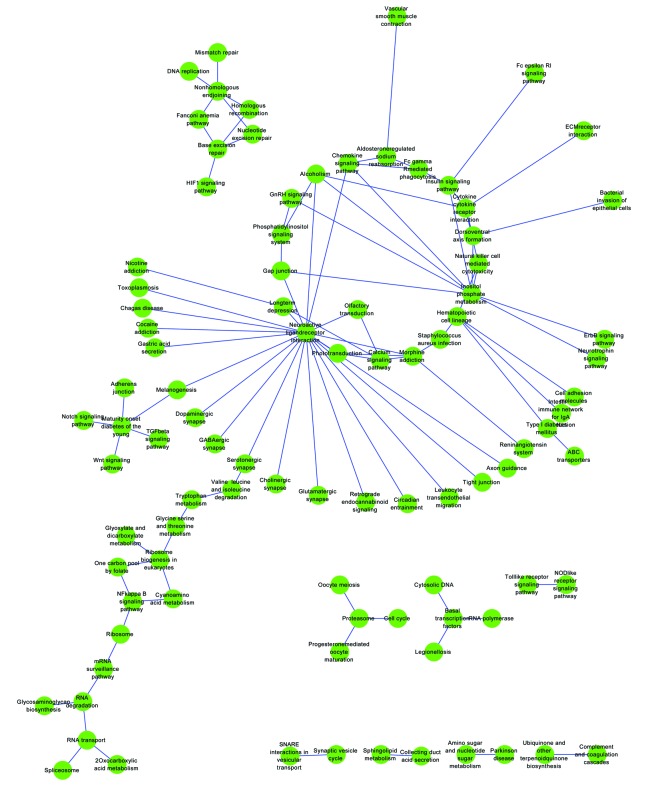
Co-change network for pathways in osteoporosis. The nodes and edges represent the pathways and pathway-pathway interactions, respectively.

A total of 227 pathways were annotated for the PPI network and the pathway-pathway interactions with P<0.01 were identified in order to construct the co-change network for pathways (Fig. 2). The co-change pathways with degree ≥4 are shown in Table II. The pathway with the degree of 22 in the constructed co-change network was neuroactive ligand receptor interaction (Table II). Other pathways were inositol phosphate metabolism (degree, 9), cytokine receptor interaction (degree, 5), hematopoietic cell lineage (degree, 5), the calcium signaling pathway (degree, 4) and the chemokine signaling pathway (degree, 4).

### Construction of a transcriptional regulatory network for DEGs

**Figure 3:**
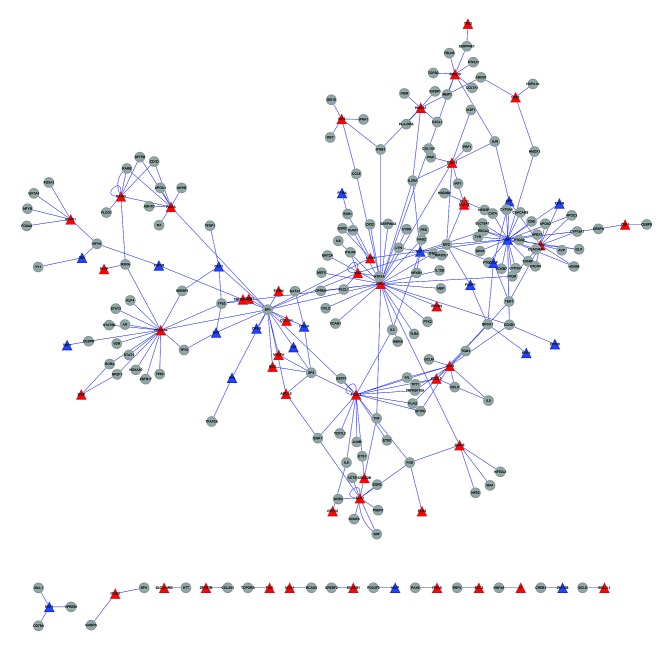
Transcriptional regulatory network for DEGs. The red, blue and grey nodes, and grey edges represent the upregulated DEGs, downregulated DEGs, other genes and transcriptional regulation associations, respectively. DEGs, differentially expressed genes.

A transcriptional regulatory network for DEGs was constructed according to the information included in the TRANSFAC database (Fig. 3). Genes with degrees ≥9 in the transcriptional regulatory network are listed in Table III. The nine genes with high degrees in the constructed transcriptional regulatory network were REL-associated protein (RELA), upstream stimulatory factor 1 (USF1), Sp1, Fos-related antigen 1 (FOSL1), cyclin-dependent kinase inhibitor 1A (CDKN1A), upstream stimulatory factor 2 (USF2), ETS domain-containing protein Elk-1 (ELK1), JUND and retinoic acid receptor α (RARA), with degrees of 29, 27, 19, 18, 17, 13, 11, 11 and 9, respectively (Table III). From the transcriptional regulatory network, Sp1 was shown to have transcriptional regulatory associations with FOSL1, RELA and CDKN1A.

## Discussion

The polymorphisms of a number of genes, including vitamin D receptor, estrogen receptor α, estrogen receptor β, LRP5 and SOST, are associated with a risk of developing osteoporosis (26,27). In the present study, the gene expression profiles of hMSCs samples from elderly patients suffering from osteoporosis and control samples were downloaded. A total of 1,127 DEGs, including 554 upregulated and 573 downregulated DEGs, were screened. Functional and pathway enrichment analyses revealed a significant involvement of DEGs in the pathways of focal adhesion and lysosome. Focal adhesion kinase may regulate the realignment of hMSCs, which is induced by mechanical stretch (28). A number of genes in the focal adhesion family have been reported as candidate genes for osteoporosis (29). Therefore, the signaling pathway of focal adhesion is likely to be important in the pathogenesis of osteoporosis.

The DEGs that were involved in the two significantly enriched pathways were used to construct a PPI network. The gene with degree of 135 in the constructed protein-protein interaction network was CTNNB1. Wnt/β-catenin signaling is involved in the anabolic response to mechanical stimulation, and bone mass accrual and maintenance. In addition, β-catenin has been shown to regulate osteoblast survival and differentiation (10,30,31). Ablation of β-catenin may promote the differentiation of osteoclast precursors into bone-resorbing osteoclasts, ultimately leading to osteoporosis (32). The results of the current study also showed that CTNNB1 and CAV1 were significantly downregulated in the osteoporosis samples, while SHC1, AKT1 and FLNA were upregulated.

The pathway with the degree of 22 in the constructed co-change network was neuroactive ligand receptor interaction. The inositol phosphate metabolism, cytokine receptor interaction, hematopoietic cell lineage, calcium signaling and chemokine signaling pathways also had relatively high numbers of interactions. It has been reported that mechanical loading may lead to an increase in the intracellular calcium concentration in osteoblasts, resulting in the activation of AKT, which is responsible for osteoblast survival and proliferation (33). Certain chemokines are essential for bone metabolism, such as osteopontin, which has been reported to be involved in the pathogenesis of osteoporosis (34). Therefore, the pathway of neuroactive ligand receptor interaction may be important in the development of osteoporosis.

The genes with high degrees in the constructed transcriptional regulatory network, were RELA, USF1, SP1, FOSL1, CDKN1A, USF2, ELK1, JUND and RARA. Activation of liver X receptor upregulates the expression of osteoclast/macrophage-related markers, including USF1/2, which has the potential to inhibit the differentiation of bone marrow-derived osteoclast precursors into osteoclasts (35). Furthermore, Sp1 had associations with the transcriptional regulation of FOSL1, RELA and CDKN1A. The collagen type I α1 and Sp1 polymorphisms are associated with reduced bone density and osteoporosis (36). Overexpression of Fos-related antigen 1, encoded by FOSL1, may increase bone formation and accelerate osteoblast differentiation in mice (37). Therefore, FOSL1, RELA and CDKN1A may also be also involved in the pathogenesis of osteoporosis.

In conclusion, the significant DEGs identified in the constructed PPI network and transcriptional regulatory network may provide useful information on the pathogenesis of osteoporosis. However, the present study did not analyze hMSCs from patients of different ages and genders. Furthermore, the results of the study require confirmation by experimental research. Therefore, the molecular mechanism underlying the development of osteoporosis demand further exploration.

## Figures and Tables

**Table I:** Five differentially expressed genes with degrees >60 in the constructed protein-protein interaction network.

Symbol	Gene ID	Degree	Type
CTNNB1	1499	135	Downregulated
SHC1	6464	117	Upregulated
AKT1	207	117	Upregulated
CAV1	857	73	Downregulated
FLNA	2316	63	Upregulated

CTNNB1, β-catenin; SHC1, SHC-transforming protein 1; AKT1, RAC-α serine/threonine-protein kinase; CAV1, caveolin 1; FLNA, Filamin A.

**Table II:** Ten pathways with degrees ≥4 in the constructed co-change network for pathways.

Pathway	Degree
Neuroactive ligand receptor interaction	22
Inositol phosphate metabolism	9
Nonhomologous end joining	5
Cytokine receptor interaction	5
Hematopoietic cell lineage	5
Morphine addiction	5
Maturity onset diabetes of the young	5
Base excision repair	4
Calcium signaling pathway	4
Chemokine signaling pathway	4

**Table III:** Nine genes with degrees ≥9 in the constructed transcriptional regulatory network.

Symbol	Gene ID	Degree
RELA	5970	29
USF1	7391	27
SP1	6667	19
FOSL1	8061	18
CDKN1A	1026	17
USF2	7392	13
ELK1	2002	11
JUND	3727	11
RARA	5914	9

RELA, REL-associated protein; USF1, upstream stimulatory factor 1; Sp1, specificity protein 1; FOSL1, FOS-related antigen 1; CDKN1A, cyclin-dependent kinase inhibitor 1A; USF2, upstream stimulatory factor 2; ELK1, ETS domain-containing protein Elk-1; RARA, retinoic receptor α.
